# Feeding Behaviour and Faecal Particle Size in Lambs Fed Concentrate-Based Diets with Grass and Maize Silages of Various Feed Values

**DOI:** 10.3390/ani16213364

**Published:** 2026-10-25

**Authors:** Carl Helander, Konstantinos Zaralis, Peder Nørgaard

**Affiliations:** 1 Department of Applied Animal Science and Welfare, Swedish University of Agricultural Sciences, P.O. Box 234, 532 23 Skara, Sweden; 2 Department of Agriculture, School of Agricultural Sciences, University of Western Macedonia, Kontopoulou str., 53100 Florina, Greece; 3 Department of Veterinary and Animal Sciences, University of Copenhagen, Grønnegårdsvej 3, 1870 Frederiksberg C, Denmark

**Keywords:** chewing behaviour, faecal particles, maize silage, rumination, sheep, sorting

## Abstract

Maize silage is increasingly used for ruminants in Nordic countries. We evaluated effects of maize maturity at harvest and maize silage inclusion rate on intake, dietary selection, chewing behaviour and faecal particle size in lambs fed concentrate-based diets. Forty crossbred ram lambs were housed in pairs (five pens per treatment) in a 2 × 2 factorial arrangement. Diets contained 58% concentrate and 42% forage (dry matter (DM) basis); forage was early- (dough stage) or late-harvested (dent stage) maize silage, alone or mixed 50:50 with grass silage. After approximately three weeks on the diets, data were collected over four days, including 24 h of video-recorded behaviour. Daily DM and neutral detergent fibre (NDF) intakes were not affected by diet. Refusals contained more NDF, more particles of 8–19 mm and <1 mm and fewer particles of 1–8 mm than the offered feed; selection for 1–8 mm particles was greatest with late-harvested maize as the sole forage. Lambs fed maize silage as sole forage spent less time eating (daily and per kg DM intake) and had fewer faecal particles <0.212 mm and more of 0.212–0.500 mm than lambs fed grass–maize diets. Maturity and inclusion rate had limited effects on intake and feeding behaviour.

## Simple Summary

During wintertime in Nordic countries, sheep are housed indoors for several months. Traditionally, they are fed grass hay or silage. To be able to produce fresh lamb meat all year round, the production system with lambing indoors during early winter months has become common practice in Swedish sheep farming. Owing to the development of maize hybrids suited to the Nordic climate, maize is now a common crop for ruminants in Sweden and other Nordic countries. Forage maize has been used in dairy production, and more recently, it is also being used in lamb production. In this study, lambs born indoors during mid-winter were fed diets in which maize silage, harvested at two stages of maturity, made up half or all of the forage. To explore wider use of forage maize in lamb production and, thereby, the resilience of Nordic sheep farmers in a changing climate, we studied the lambs’ feed intake, selection among feed particles, eating and rumination behaviour and the size of particles in their faeces.

## 1. Introduction

In Sweden, the area cultivated with maize increased from approximately 17,000 ha in 2015 to 37,700 ha in 2025 [1]. Hybrids adapted to high-latitude growing conditions have enabled forage maize production in Nordic environments [2]. During grain filling, starch accumulates in the kernels as maize progresses towards physiological maturity [3]. Whole-crop maize harvested for optimal feed value usually contains less than 100 g crude protein (CP) per kg DM [2], which is less than typical grass silage. Supplementary protein may therefore be required, depending on the composition of the complete diet and the protein requirements of growing lambs.

Inclusion of maize silage in the diet has been shown to maintain or increase feed intake in sheep when partially replacing grass silage, without affecting animal performance [4,5]; similar results have been observed when replacing grain or forage sorghum silage [6] or whole-crop barley silage [7] with maize silage. When comparing maturity stages, Keady and Hanrahan [8] showed that feed intake and growth rate of finishing lambs were not affected by feeding maize silage containing 28% or 3% starch on a dry matter (DM) basis. In the larger experiment from which the present data originate, maize silage harvested at either maturity stage could replace grass silage in diets for high-producing lambs [9]. Although these studies provide information on feed intake and growth, they do not explain how lambs respond to the physical characteristics of maize silage-based diets. Small ruminants can select among feed fractions with different physical and chemical characteristics [10], so the composition of the diet consumed may differ from that of the ration offered. Understanding this selection is therefore relevant to interpreting fibre intake and chewing behaviour. How growing lambs select particles from concentrate-based total mixed rations (TMRs) and how their eating behaviour, rumination and faecal particle-size distribution respond to maize silage maturity and to maize silage inclusion as the sole forage or in combination with grass silage remain insufficiently understood. Such data may contribute to the further development of feed evaluation systems for growing lambs by improving our understanding of how forage characteristics influence dietary selection, chewing behaviour and faecal particle-size distribution. The present study addresses these behavioural and physical responses through complementary measurements from the lamb experiment reported by Helander et al. [9].

Forage characteristics, such as NDF content and digestibility, and the dietary forage proportion affect intake and chewing activity [11,12]. In cattle, time spent chewing is closely related to NDF intake, the proportion of indigestible NDF (iNDF) and feed particle size [13,14], and increasing lignification during forage maturation can reduce the digestibility of cell-wall material [15]. In growing steers, advancing maturity of whole-crop barley silage increased selection for starch, rumination time per kg NDF intake and faecal particle size [16], and in dairy cows, inclusion of maize silage in the diet increased the concentration of undigested feed particles and NDF in faeces [17]. Evidence from sheep indicates that forage quality and maturity can likewise influence chewing behaviour and faecal particle-size distribution [18,19], and a cross-species analysis including sheep related faecal particle size to body weight, forage lignification, forage NDF intake and the concentrate-to-forage ratio [20]. These findings provide a rationale for examining maize-based diets in growing lambs, while recognising that results from cattle or other forage types cannot be transferred directly. Faecal particle size is closely related to the size of the particles leaving the reticulo-rumen [21]; faecal particle-size distribution therefore provides indirect information on particle breakdown and passage, but it does not directly measure rumen function or fibre digestibility.

As maize matures, grain filling increases the contribution of starch-rich kernels to the whole crop [3], while increasing lignification may reduce the digestibility of the vegetative tissues [15]. A greater contribution of kernels could reduce the physical limitation of intake imposed by fibrous forage, providing a basis for predicting increased feed intake with advancing maize maturity. Greater differences between starch-rich kernel fragments and fibrous stem and leaf particles, together with a higher DM concentration, could also facilitate selective consumption [10], and the more lignified stover could prolong rumination and increase faecal particle size, as observed with advancing maturity of whole-crop barley silage in steers [16]. Increasing the proportion of maize silage at the expense of grass silage changes both the chemical composition and the physical characteristics of the forage fraction. A lower contribution of physically effective forage fibre could reduce the stimulation of rumination and the physical limitation of intake, providing a basis for predicting decreased rumination time and increased feed intake with increasing maize silage inclusion; higher intake and more opportunities for selection could in turn prolong eating time. Because grass silage dilutes the maize-derived particles in the ration, the effect of maize maturity may also depend on how much maize contributes to the forage fraction. A 2 × 2 factorial arrangement was therefore used to evaluate whether the response to maize maturity differed when maize silage made up half or all of the forage DM.

Based on these considerations, we hypothesised that advancing maize maturity at harvest would increase feed intake, dietary selection, rumination time and faecal particle size, while leaving eating time unaffected. We further hypothesised that increasing the proportion of maize silage in the forage would increase feed intake, dietary selection, eating time and faecal particle size, while decreasing rumination time. In addition, we evaluated whether the response to maize maturity depended on the maize silage inclusion rate.

## 2. Materials and Methods

All experimental methods used in this experiment were approved by the Gothenburg Regional Animal Ethics Committee (approval number 265-2009). The experiment was carried out at the Götala Beef and Lamb Research Centre (58°22′ N, 13°29′ E, altitude 120 m above sea level), Swedish University of Agricultural Sciences (SLU), Skara, Sweden.

### 2.1. Experimental Forages

The maize crop (sown on 20 April 2010) was harvested at the dough stage of maturity on 14 September and at the dent stage of maturity on 12 October 2010, corresponding to approximately 28% and 36% whole-crop DM at harvest, respectively. These stages were chosen to represent an early harvest and the later harvest time commonly practised for forage maize fed to ruminants in Sweden. The analysed early- and late-harvested maize silages contained 265 and 381 g DM kg^−1^ and 230 and 362 g starch kg^−1^ DM, respectively, and the grass silage contained 366 g DM kg^−1^, 137 g CP kg^−1^ DM and 554 g NDF kg^−1^ DM. The chemical composition and fermentation characteristics of the silages are presented in Table 1. At harvest, forage maize was chopped to a length of cut of 14 mm and ensiled in round bales with 2 litres per ton herbage of the chemical additive Kofasil^®^ Stabil (sodium benzoate 250 g/L and potassium sorbate 150 g/L; Addcon Europe GmbH, Bonn, Germany) and wrapped with ten layers of plastic film. The grass silage (mainly perennial ryegrass, timothy and meadow fescue) was harvested as the first regrowth, chopped to 25 mm and ensiled in a bunker silo with the additive Promyr^®^ NT570 (50.0% formic acid, 17.1% propionate and 5.6% sodium; Perstorp AB, Perstorp, Sweden).

### 2.2. Animals

In the experiment, 40 twin- or triplet-born crossbred ram lambs were selected from a local sheep herd at weaning at 66 ± 9.4 (mean ± SD) days of age. The lambs were offspring of Finewool × Dorset ewes sired by a Texel ram.

### 2.3. Experimental Design and Feeding

The experiment comprised four dietary treatments in a 2 × 2 factorial arrangement, with maize maturity stage at harvest (early or late) and maize silage inclusion rate (50% or 100% of forage DM) as factors. On a forage DM basis, the four treatments were as follows: (1) 50% early-harvested maize silage and 50% grass silage (E50), (2) 100% early-harvested maize silage (E100), (3) 50% late-harvested maize silage and 50% grass silage (L50), and (4) 100% late-harvested maize silage (L100). All diets contained 58% concentrate and 42% forage on a DM basis. The concentrate consisted of dried distiller’s grains plus solubles (DDGS), rolled barley and heat-treated rapeseed meal in varying proportions.

The 40 lambs were housed in 20 pens (6.0 m^2^, with wheat-straw bedding), with two lambs per pen and five pens per treatment; thus, no treatment was housed in a single pen. At the start of the larger feeding experiment, the lambs were divided into four homogeneous groups of ten lambs on the basis of initial body weight (BW), age and number of siblings, and the groups were randomly assigned to the four dietary treatments, as described by Helander et al. [9]. Within each treatment, the ten lambs were housed in five pens of two lambs. The pen was the experimental unit for feed intake, dietary selection and faecal particle size, whereas behaviour was recorded for the individual lambs within each pen.

The experiment was part of a larger study [9]. The lambs had been fed the experimental diets for approximately three weeks (adaptation period) before the 4-day data-collection period (days 1–4) of the present study started, during which feed intake was recorded, feed, refusals and faeces were sampled, and behaviour was video-recorded. At the start of the experiment, the lambs weighed 31 ± 2.8 (mean ± SD) kg, when averaged over treatments. The diets were formulated for equal contents of metabolisable energy (ME), CP, and vitamins and minerals per kg DM intake and fed as total mixed ration (TMR; Table 1 and Table 2). In the following text, the diets containing both maize and grass silage (E50 and L50) will be referred to as grass–maize diets and the diets containing maize silage as the sole forage (E100 and L100) as maize diets.

The lambs were offered the experimental diets *ad libitum* once daily at approximately 10:00 h, allowing for about 20% refusals. The TMRs for the 4-day period were mixed on day 1, immediately before the first feeding. Refusals were collected and the feed troughs were emptied each day before fresh TMR was offered, i.e., approximately 24 h after the previous feeding. All animals had free access to salt and water throughout the whole experiment.

### 2.4. Recording and Samplings

Behaviour of each lamb was recorded by continuous video recording, using three high-resolution cameras per pen, during the four consecutive days. Eating, rumination, standing and lying were registered from 06:00 h on day 3 to 06:00 h on day 4 by instantaneous scan sampling of the recordings [22]. Before the experiment, the scan interval was evaluated in a pilot recording of two lambs from the same flock fed a similar diet. The recordings were decoded at 5 min intervals over a complete 24 h period (288 scans per lamb), and a 10 min data set was obtained by retaining every second scan (144 scans per lamb). Compared with the 5 min scans, the 10 min scans changed the estimated daily durations by at most 10 min/day for eating (≤4%), rumination (≤2%), lying without ruminating (≤1%) and standing (≤6%), whereas the number of bouts was underestimated (by 1–3 bouts/day for eating, 2–3 for rumination, 4–6 for lying and 7–9 for standing) and bout length was correspondingly overestimated. The recordings in the present experiment were therefore scanned at 10 min intervals, giving 144 scheduled observations per lamb. The lambs were assumed to perform the same behaviour during the 10 min between two consecutive scans, and the daily duration of each behaviour was estimated as the proportion of scans assigned to that behaviour multiplied by 1440 min. Eating, ruminating while lying, ruminating while standing, lying without ruminating, and standing without eating or ruminating were recorded as mutually exclusive categories. Each lamb was individually marked with sheep marking spray on both sides and on the back, and the three cameras per pen allowed continuous identification of both lambs in each pen. Chewing times per kg DMI and per kg NDF intake were calculated for each lamb from the mean intake per lamb in its pen. Instantaneous sampling at 10 min intervals accurately estimates lying time in feedlot lambs, whereas shorter intervals are recommended for brief behaviours such as feeding and standing [23]; some short bouts may therefore have been missed, and a single 24 h observation period does not quantify day-to-day variation (see Section 4).

Feed offered and refusals were recorded per pen. Feed intake and dietary selection were therefore calculated per pen, with five pens per treatment; individual intake was not measured. Where intake is expressed per lamb, pen intake was divided by two (the number of lambs per pen). The lambs were weighed individually on the last day of the 4-day recording period.

The TMRs offered were weighed and sampled daily for each treatment, and refusals were weighed and sampled daily from each pen. The daily samples of offered TMR and refusals were pooled into one TMR sample per treatment and one refusal sample per pen, respectively. Silages and concentrates were sampled daily and pooled into one composite sample per silage and per concentrate, respectively. Fresh faeces (approximately 100 g per occasion) were collected from the bedding surface of each pen twice daily during the 4-day collection period. Only freshly voided, still body-warm faeces without visible contamination by bedding material or urine were sampled. The eight samples from each pen were pooled into one composite sample per pen; the faecal samples therefore represented the pen and were not attributed to individual lambs. The samples of silages, concentrates, TMRs, refusals and faeces were kept at −25 °C until analysis.

### 2.5. Particle-Size Analysis of TMRs, Refusals and Faeces

Pooled samples from the 4-day period of offered TMRs and refusals were separated into particle-size fractions using the Penn State Particle Separator (Nasco, Fort Atkinson, WI, USA), as described by Heinrichs and Kononoff [24], with an additional sieve with a pore size of 30 mm. Particles retained on the 30, 19, 8 and 1 mm sieves and in the bottom pan correspond to the fractions >30, 19–30, 8–19, 1–8 and <1 mm, respectively, and the proportion of each fraction was calculated relative to the total sample weight. Dietary selection for each fraction was calculated for each pen as the proportion of the fraction in the refusals minus its proportion in the offered TMR (percentage units); negative values indicate preferential consumption and positive values rejection of the fraction. For each analysis, approximately 300 g of sample was placed on the top sieve and shaken horizontally in eight sets of five shakes, with the separator rotated a quarter turn between sets [24]; proportions were calculated on an as-fed basis.

Twelve grams of faeces were divided into three subsamples of 4 g each and washed in nylon bags with a pore size of 0.01 mm, with a small amount of detergent added to each bag, as described by [20,25]. The nylon bags were then emptied, and the samples were freeze-dried. The dry particles were separated into six fractions by dry-sieving with a vertical sieve shaker (Retsch AS 200; Retsch GmbH, Haan, Germany), using sieves of 2.360, 1.000, 0.500, 0.212 and 0.106 mm aperture and a bottom bowl, as described by [19,25]. Sieving lasted 5 min; thereafter, each fraction was checked for clustered particles, which were separated by gentle manual brushing before an additional 1 min sieving. This was repeated until no clustered particles remained. The arithmetic mean particle size (APS) and the geometric mean particle size (GPS) were estimated according to [26].

### 2.6. Chemical Analyses

Silages, TMRs and refusals were analysed for contents of DM, NDF, acid detergent fibre (ADF), acid detergent lignin (ADL) and ash. The NDF was analysed using the ANKOM220 fibre analyser (Ankom Technology, Macedon, NY, USA) according to Van Soest et al. [27], with heat-stable α-amylase included and sodium sulphite omitted from the analysis. The NDF, ADF and ADL values are expressed on an ash-free basis. Ash content was determined by incineration at 550 °C for 16 h. Silage samples for iNDF analysis were milled through a 1.5 mm screen in a Wiley mill, and iNDF was determined after a 288 h in situ incubation [28].

Total nitrogen (N) content of silages and TMRs was analysed using the Kjeldahl procedure, and CP was calculated as total N × 6.25. Refusals were not analysed for CP or starch; intakes of CP and ME were therefore calculated from DMI and the CP and ME concentrations of the offered TMR, whereas intakes of NDF, ADF and ADL were calculated from the amounts and concentrations in offered feed and refusals. Consequently, dietary selection could be evaluated only for NDF, ADF and particle-size fractions. The metabolisable protein (MP) was calculated according to [29]. The in vitro organic matter digestibility (IVOMD) of the grass silage was analysed according to [30], and the ME was calculated from IVOMD according to [31]. Because this method and the associated ME equation were calibrated for ley forages, the IVOMD of the maize silages was instead analysed by the two-stage technique [32], and the ME values were calculated according to [33]. The ME values were used for diet formulation and are calculated estimates rather than measurements of in vivo energy utilisation. Starch (including maltodextrins) was determined enzymatically according to [34]. Water-soluble carbohydrates (WSCs) were determined by the anthrone method [35]. The pH was potentiometrically determined using a calibrated pH electrode. Lactic acid was analysed by HPLC [36]. Volatile fatty acids were determined by gas chromatography [37]. Ammonia concentration was colorimetrically determined by use of a continuous flow analyser (Skalar Analytical B.V., Breda, The Netherlands).

### 2.7. Statistical Analysis

Data were analysed as a 2 × 2 factorial arrangement of treatments in a completely randomised design, using the MIXED procedure of SAS (release 9.3; SAS Institute Inc., Cary, NC, USA). The model included the fixed effects of maize silage inclusion rate (I; 50% or 100% of forage DM), maize maturity stage at harvest (M; early or late) and the I × M interaction. Feed intake, dietary selection and faecal particle size were recorded at pen level, and the pen was the experimental unit (n = 5 pens per treatment). Chewing and other behavioural data were recorded for individual lambs and analysed with lamb nested within pen as a random effect, so that the pen remained the experimental unit for testing treatment effects (n = 5 pens and 10 lambs per treatment). Differences between offered feeds and refusals in the contents of NDF and ADF and in the proportions of particles retained on each sieve of the Penn State Particle Separator were analysed with the GLM procedure of SAS using treatment means (n = 4), because only one pooled sample of offered TMR was available per treatment; this comparison therefore has low statistical power and is interpreted with caution.

Because each factor had two levels, main effects were evaluated directly by the F-tests. Pairwise comparisons between treatment least-squares means, adjusted according to Tukey, were made only when the I × M interaction was significant (*p* < 0.05). Effects were considered significant at *p* < 0.05, and 0.05 ≤ *p* < 0.10 was regarded as a tendency; tendencies were not followed by post hoc comparisons. Exact *p*-values are presented in the result tables.

## 3. Results

### 3.1. Feed Intake

The daily ADF intake tended to increase by including grass silage in the diets (*p* = 0.06). Neither daily dry matter intake (DMI) nor NDF intake (NDFI) was affected by maturity stage or inclusion rate of maize silage (*p* > 0.10). The BW of the lambs was similar in all treatments (*p* > 0.10; Table 3).

### 3.2. Dietary Selection

**Figure 1:**
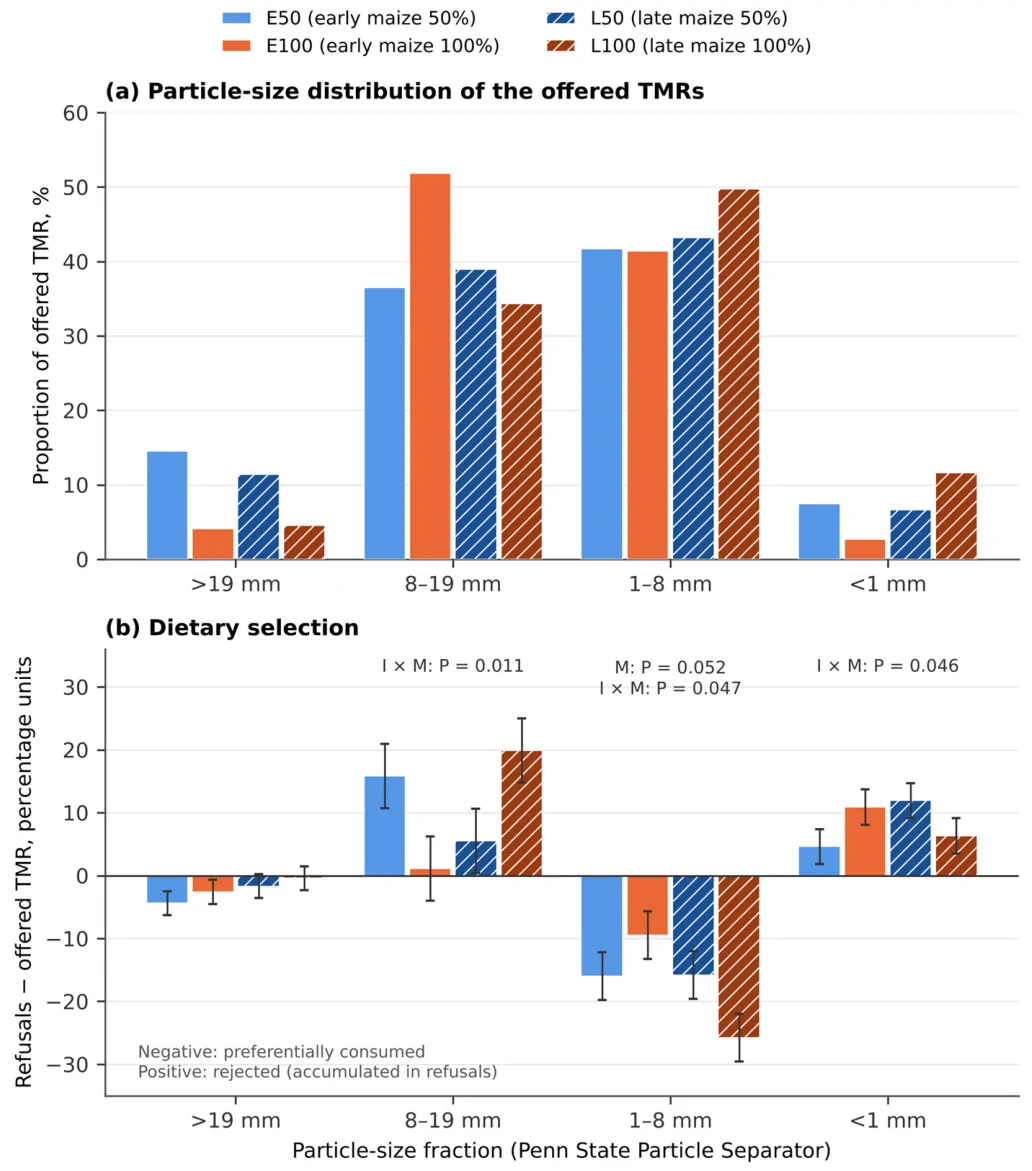
(**a**) Particle-size distribution of the offered TMRs (one pooled sample per treatment) and (**b**) dietary selection for each particle-size fraction, calculated as the proportion of the fraction in the refusals minus its proportion in the offered TMR (percentage units; means ± SEM, n = 5 pens per treatment). Negative values indicate preferential consumption, and positive values represent rejection of the fraction. Fractions: >19 mm (particles retained on the 19 and 30 mm sieves), 8–19 mm (8 mm sieve), 1–8 mm (1 mm sieve) and <1 mm (bottom pan) of the Penn State Particle Separator. E50 and E100, early-harvested maize silage as 50% or 100% of forage DM; L50 and L100, late-harvested maize silage as 50% or 100% of forage DM; I, inclusion rate; M, maturity stage.

Selection for particles retained on the 1 mm sieve (1–8 mm) depended on the combination of maize inclusion rate and maturity stage (I × M, *p* = 0.047; Figure 1b). With maize silage as the sole forage, late harvest increased the selection for this fraction compared with early harvest (−25.8 vs. −9.5 percentage units; L100 vs. E100, *p* = 0.038 after Tukey adjustment), whereas no difference between maturity stages was found for the grass–maize diets (−15.8 vs. −16.0 percentage units). Averaged over inclusion rates, the selection for this fraction tended to be greater for late- than for early-harvested maize silage (*p* = 0.052). There were also I × M interactions for the rejection of particles retained on the 8 mm sieve (*p* = 0.011), which was greatest for L100 and E50, and of particles in the bottom pan (*p* = 0.046), which was greatest for E100 and L50, but none of the pairwise differences between treatments was significant after Tukey adjustment. No other treatment effects on dietary selection were found.

Averaged over the four diets, the proportions of particles retained on the 30 and 19 mm sieves did not differ between offered feeds and refusals. The proportion of particles retained on the 8 mm sieve was 26% greater (*p* < 0.01), and that in the bottom pan was 2.2 times greater (*p* < 0.001) in the refusals than in the offered feeds. In contrast, the proportion of particles retained on the 1 mm sieve was 62% greater in the offered feeds than in the refusals, when averaged over the four diets (*p* < 0.001; Figure 1).

Averaged over maize inclusion rates and maturity stages at harvest, the NDF content was 19% higher (383 vs. 323 g kg^−1^ DM) and the ADF content was 26% higher (203 vs. 160 g kg^−1^ DM) in the refusals than in the offered feeds (*p* < 0.001).

### 3.3. Chewing Behaviour

Lambs fed the maize diets spent 35 min less time eating per day (*p* = 0.040) and 40 min less time eating per kg DMI (*p* = 0.027), and tended to spend 88 min less time eating per kg NDFI (*p* = 0.087), than lambs fed the grass–maize diets. The number of eating bouts per day was affected neither by inclusion rate nor by maturity stage of the maize at harvest (*p* > 0.10). The eating bouts were 3.3 min shorter for the lambs fed the maize diets than for those fed the grass–maize diets (*p* = 0.012) and tended to be 2.4 min longer for the lambs fed the late-harvested than for those fed the early-harvested maize silage, when averaged over inclusion rates (*p* = 0.06; Table 4).

Lambs tended to spend 74% more time ruminating in a standing position (*p* = 0.08) when fed maize diets than when fed grass–maize diets, and inclusion of grass silage in the diet tended to increase the rumination time per kg DMI by 33 min (*p* = 0.07). Total daily chewing time per kg DMI was 72 min longer in grass–maize diets than in maize diets, averaged across maturity stages (*p* = 0.020; Table 4).

The inclusion of grass silage in the diets decreased the number of standing bouts by 1.3 (*p* = 0.045) and tended to decrease the daily time spent standing, including the time spent standing while eating and ruminating by 36 min (*p* = 0.07). There was an I × M interaction for the length of lying bouts without ruminating (*p* = 0.049), which were longest for L100 (30.2 vs. 25.1–26.7 min), although none of the pairwise differences was significant after Tukey adjustment. Increased maturity stage of maize tended to decrease the daily time spent lying down, including time spent ruminating lying down, by 34 min (*p* = 0.08; Table 4).

**Figure 2:**
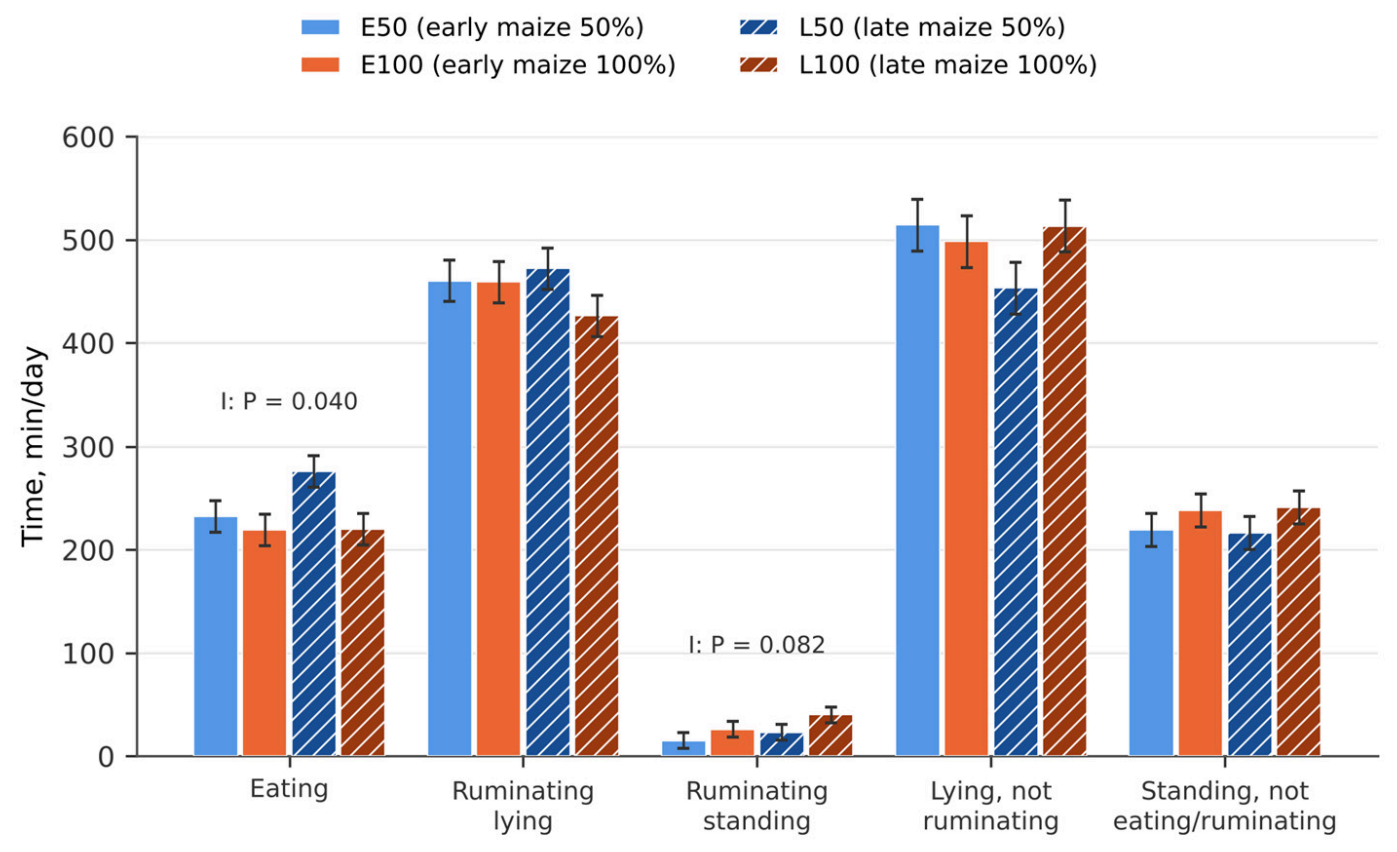
Daily time (min/day; means ± SEM, n = 10 lambs in five pens per treatment) spent eating, ruminating while lying, ruminating while standing, lying without ruminating, and standing without eating or ruminating. The five categories are mutually exclusive and sum to 1440 min. E50 and E100, early-harvested maize silage as 50% or 100% of forage DM; L50 and L100, late-harvested maize silage as 50% or 100% of forage DM; I, effect of inclusion rate (Table 4).

The distribution of daily time spent on eating, chewing, lying and standing behaviours is shown in Figure 2. Averaged across maturity stages, lambs fed the grass–maize diet spent relatively more of their daily time eating than lambs fed the maize diet (0.175 vs. 0.150, *p* = 0.040).

### 3.4. Faecal Particle Size

Feeding grass–maize diets increased the proportion of faecal particles smaller than 0.212 mm compared with maize diets (37.0 vs. 30.7%), owing to greater proportions of particles retained on the 0.106 mm sieve (*p* = 0.026) and in the bottom bowl (*p* = 0.032). The maize diets increased the proportion of faecal particles between 0.212 and 0.500 mm by 8% (53.3 vs. 49.3%; *p* = 0.022) and tended to increase the proportions of particles between 0.500 and 1.000 mm (*p* = 0.092) and larger than 2.360 mm (0.20 vs. 0.05%; *p* = 0.096) compared with the grass–maize diets. The arithmetic and geometric mean particle sizes were not affected by inclusion rate or maturity stage (*p* > 0.10; Table 5).

## 4. Discussion

In the present study, neither maize maturity at harvest nor maize silage inclusion rate affected daily DM or NDF intake. The main treatment effects concerned eating time and faecal particle-size distribution: lambs fed grass–maize diets spent more time eating, both daily and per kg DMI, and had a larger proportion of fine faecal particles than lambs fed maize silage as the sole forage. The effect of maize maturity on the selection for particles of 1–8 mm depended on the maize silage inclusion rate and was evident only when maize silage was the sole forage, whereas maturity had limited effects on chewing behaviour and faecal particle size. The two maize silages were of high feed value, and their DM contents (265 and 381 g kg^−1^) bracketed the range of 30–35% that is commonly considered optimal for harvest of the maize crop [38]. The grass silage was representative of silages offered to sheep in the Nordic countries in terms of DM, ME, CP and NDF contents, and the daily DMI of the lambs was similar to that in our previous studies with grass silage as the only forage [39,40]. The results should therefore be interpreted in the context of high-quality silages and balanced, high-concentrate diets.

The higher NDF and ADF concentrations in refusals than in offered feeds show that the lambs selectively rejected fibre-rich ration fractions, in agreement with results for ewes fed TMRs based on grass silage [41]. Dietary sorting is common in ruminants, and particularly in small ruminants [10]. Compared with the offered feeds, the refusals also contained greater proportions of particles retained on the 8 mm sieve (8–19 mm) and in the bottom pan (<1 mm) and a smaller proportion of particles retained on the 1 mm sieve (1–8 mm), and the selection for the 1–8 mm fraction was greatest for L100. This interaction is consistent with the rationale that the effect of maize maturity depends on the contribution of maize to the forage fraction: when late-harvested maize was the sole forage, the offered TMR contained the largest proportion of 1–8 mm particles (Figure 1a), which were preferentially consumed, whereas the inclusion of grass silage may have diluted this effect. In whole-crop cereal silages, selection for starch-rich kernels at the expense of fibrous stover has been reported, particularly at advanced maturity [16], and the 1–8 mm fraction is likely to contain kernel fragments as well as concentrate particles. The greater proportion of fine particles in refusals may partly reflect fine material that accumulated at the bottom of the troughs rather than active rejection. However, botanical fractions were not separated and starch was not analysed in refusals in the present study, so these observations do not establish preferential consumption of maize kernels, leaves or individual concentrate ingredients.

The particle-size distributions of the offered diets also differed (Figure 1). The grass silage was chopped to a longer length (25 mm) than the maize silages (14 mm), which is consistent with the larger proportion of particles retained on the 19 and 30 mm sieves in the grass–maize diets. The larger proportions of particles retained on the 1 mm sieve and in the bottom pan in diets with late-harvested maize silage are consistent with the higher kernel (starch) content of the more mature crop (Table 1). The proportions of the concentrate ingredients also differed between diets (Section 2.3) and may have contributed to these differences.

The higher DM content of the TMRs containing late-harvested maize silage (553 and 582 vs. 527 and 493 g kg^−1^; Table 2) may have facilitated particle separation and selective feeding, because drier rations are less cohesive. However, the relationship between ration DM content and sorting was not tested in the present experiment, and DM content should therefore be regarded as a possible contributing factor rather than an established explanation. Chewing time and faecal particle-size distribution were not affected by maturity stage at harvest of the maize. In the larger study of which this experiment was a part, maize silage harvested at either maturity stage could replace grass silage in diets for high-producing lambs; however, effects on intake and carcass traits differed between years [9].

Forage NDF [11,13] and forage iNDF [14] have previously been identified as major determinants of chewing time in ruminants. In the present study, the inclusion of grass silage in the diet increased daily eating time, as well as eating and total chewing times per kg DMI (*p* < 0.05), and tended to increase rumination time per kg DMI (*p* = 0.07; Table 4). The grass silage had higher NDF and iNDF concentrations (554 g NDF kg^−1^ DM; 100 g iNDF kg^−1^ NDF) than the maize silages (414 and 404 g NDF kg^−1^ DM; 74 and 83 g iNDF kg^−1^ NDF), and the grass–maize diets had higher dietary NDF and ADF concentrations (Table 2). However, the grass silage was also chopped to a longer length (25 vs. 14 mm), and the rations differed in DM content and particle-size distribution. These characteristics varied together, and their individual effects on eating and rumination cannot be separated in this experiment. The longer eating time with grass inclusion, in the absence of a detectable difference in DMI, indicates that forage source altered the time required to consume the ration. From a practical perspective, maize silage could be used as the sole forage in these balanced, high-concentrate diets without reducing DMI, but the shorter eating time and total chewing time per kg DMI indicate a lower physical structure of such diets [14], which should be considered when rations with a high proportion of concentrate are formulated; similar nutrient targets do not necessarily result in similar feeding time.

Grass–maize diets were also associated with a greater proportion of faecal particles <0.212 mm and a smaller proportion of particles between 0.212 and 0.500 mm than maize diets, whereas the arithmetic and geometric mean particle sizes did not differ (Table 5); the tendency for particles >2.360 mm concerned a fraction that made up less than 0.5% of the faecal particles. This shift towards finer faecal particles is consistent with more extensive particle breakdown during the longer chewing time per kg DMI, but the data show an association rather than a causal relationship. Faecal particle-size distribution describes the particles excreted and provides indirect information on particle breakdown and passage from the reticulo-rumen [21]; rumen fermentation, passage rate and fibre digestibility were not measured, and the results do not allow conclusions about rumen function or fibre digestibility. In a cross-species analysis, faecal particle size in sheep was related to body weight, forage lignification, forage NDF intake and the concentrate-to-forage ratio [20]. In the present study, the concentrate proportion was the same in all diets, and the ADL-to-NDF ratio was similar for the three silages (0.05–0.06; Table 1), whereas forage NDF intake was likely higher with the grass–maize diets; differences in forage NDF intake may therefore have contributed to the observed differences in faecal particle size.

Some limitations of this study should be acknowledged. Treatments were replicated in five pens per treatment, with two lambs sharing each ration, which limited the number of independent experimental units and precluded measurement of individual feed intake; chewing times per kg DMI were therefore calculated from the mean intake per lamb in each pen. The comparison of offered feeds and refusals was based on treatment means (n = 4) and therefore had low statistical power. Behaviour was assessed over a single 24 h period using 10 min scan sampling, which may have missed short bouts and does not quantify day-to-day variation. Faecal samples were collected from the bedding and pooled at pen level, which precluded individual attribution. Forage fibre characteristics, chop length, ration DM content, particle-size distribution and the proportions of concentrate ingredients were not varied independently. Bout frequency and bout length are less precise than daily durations with 10 min scans and should be interpreted with caution; because the same sampling method was used for all treatments, this is expected to affect absolute values more than treatment comparisons. Finally, faecal particle-size distribution is an indirect outcome, and rumen function and in vivo digestibility were not measured.

## 5. Conclusions

Under the conditions of this study, neither maize maturity at harvest nor maize silage inclusion rate affected daily DM or NDF intake of lambs fed high-concentrate diets. Delayed maize harvest increased the selection for feed particles of 1–8 mm when maize silage was the sole forage, but not when it was mixed with grass silage. Feeding maize silage as the sole forage decreased daily eating time and eating time per kg DMI compared with a 50:50 mixture of maize and grass silage. It also decreased the proportion of faecal particles smaller than 0.212 mm and increased the proportion of particles between 0.212 and 0.500 mm. Overall, high-feed-value maize silage harvested at either maturity stage, as the sole forage or combined with grass silage, resulted in similar feed intake and limited differences in feeding behaviour of lambs fed balanced, high-concentrate diets. These findings apply to the silages and diets evaluated here and do not establish differences or equivalence in forage digestibility.

## Figures and Tables

**Table 1:** Chemical composition of the feed stuffs used in the experiment.

Item	Early Maize Silage	Late Maize Silage	Grass Silage	DDGS ^1^	Barley	Rapeseed Expeller
DM, g kg^−1^	265	381	366	902	858	874
Ash, g kg^−1^ DM	54	44	75	44	26	72
CP, g kg^−1^ DM	104	93	137	358	119	377
NDF ^2^, g kg^−1^ DM	414	404	554	302	189	337
ADF ^3^, g kg^−1^ DM	229	215	306	-	-	-
ADL ^4^, g kg^−1^ DM	20	21	31	-	-	-
iNDF ^5^, g kg^−1^ NDF	74	83	100	-	-	-
Starch, g kg^−1^ DM	230	362	-	49	640	45
Crude fat, g kg^−1^ DM	-	-	-	69	29	13
IVOMD ^6^, g kg^−1^	>930	>930	849	-	-	-
ME ^7^, MJ kg^−1^ DM	11.0	11.2	11.0	13.7	13.3	12.4
WSC ^8^, g kg^−1^ DM	37	26	46	-	-	-
Lactic acid, g kg^−1^ DM	58	41	77	-	-	-
Acetic acid, g kg^−1^ DM	16	6	7	-	-	-
Butyric acid, g kg^−1^ DM	0	0	1.1	-	-	-
Ethanol, g kg^−1^ DM	1.9	0.7	2.4	-	-	-
Ammonia-N, g kg^−1^ N	65	62	76	-	-	-
pH	3.9	4.2	4.2	-	-	-

^1^ Dried distiller’s grain plus solubles, ^2^ neutral detergent fibre, ^3^ acid detergent fibre, ^4^ acid detergent lignin, ^5^ indigestible NDF, ^6^ in vitro organic matter digestibility, ^7^ metabolisable energy, ^8^ water-soluble carbohydrates. For DM, ash, CP, NDF, starch, crude fat, IVOMD and ME, n = 3. For WSC, acids, ethanol, ammonium and pH, n = 2.

**Table 2:** Nutrient content of the feed rations.

Item	E50 ^1^	E100 ^2^	L50 ^3^	L100 ^4^
DM, g kg^−1^ fresh matter	527	493	553	582
*per kg DM*				
ME ^5^, MJ	12.1	12.1	12.1	12.1
CP, g	200	201	201	200
MP ^6^, g	109	115	109	116
NDF ^7^, g	355	300	321	322
ADF ^8^, g	186	149	168	150
ADL ^9^, g	35	32	33	33
Starch, g	196	222	198	238
Crude fat, g	35	36	35	35
Calcium, g	7.4	7.7	7.6	7.4
Phosphorus, g	6.2	6.4	6.3	6.6

^1^ Early maize silage 50% of forage DM, ^2^ early maize silage 100% of forage DM, ^3^ late maize silage 50% of forage DM, ^4^ late maize silage 100% of forage DM, ^5^ metabolisable energy, ^6^ metabolisable protein, ^7^ neutral detergent fibre, ^8^ acid detergent fibre, ^9^ acid detergent lignin.

**Table 3:** Effects of dietary inclusion rate of maize silage (I) and maturity stage at harvest (M) on daily intake of lambs.

						Effects		
Item	E50 ^1^	E100 ^2^	L50 ^3^	L100 ^4^	SEM ^5^	I	M	I × M
*Intake*								
DM, kg day^−1^	1.3	1.4	1.3	1.4	0.08	0.277	0.620	0.966
DM, g kg^−1^ BW	35.1	37.6	35.4	38.3	2.54	0.300	0.858	0.938
NDF, g day^−1^	435	392	405	403	27.9	0.437	0.746	0.479
NDF, g kg^−1^ BW	12.0	10.9	11.0	11.1	0.95	0.596	0.662	0.533
ADF, g day^−1^	217	179	203	194	11.9	0.060	0.969	0.253
ADL, g day^−1^	45.5	44.2	44.9	51.9	3.03	0.359	0.258	0.191
CP, g day^−1^	255	272	263	279	15.2	0.285	0.622	0.959
ME, MJ day^−1^	15.4	16.4	15.8	16.9	0.92	0.285	0.620	0.972
BW, kg	36.4	36.1	37.1	36.5	1.1	0.679	0.615	0.878

^1^ Early maize silage, 50% of forage DM; ^2^ early maize silage, 100% of forage DM; ^3^ late maize silage, 50% of forage DM; ^4^ late maize silage, 100% of forage DM; ^5^ standard error of the mean. Values for I, M and I × M are *p*-values. n = 5 pens (two lambs per pen) per treatment; intake per lamb was calculated as pen intake divided by two.

**Table 4:** Effects of dietary inclusion rate of maize silage (I) and maturity stage at harvest (M) on eating, ruminating, total chewing, standing and lying behaviours of lambs.

Item	E50 ^1^	E100 ^2^	L50 ^3^	L100 ^4^	SEM ^5^	Effects		
						I	M	I × M
*Eating*								
Time								
min/day	232	219	276	220	15.4	0.040	0.163	0.182
min/kg DMI	185	164	217	158	16	0.027	0.432	0.274
min/kg NDFI	539	510	614	467	48	0.087	0.749	0.242
min/bout	18.8	16.0	21.7	17.9	1.15	0.012	0.057	0.686
Bouts, no./day	12.5	13.6	13.2	12.3	0.78	0.900	0.705	0.218
*Rumination*								
Time								
min/day total	475	485	495	466	20	0.640	0.980	0.343
min/day lying	460	459	472	426	20	0.268	0.615	0.288
min/day standing	15	26	23	40	7.5	0.082	0.164	0.696
min/bout	28.6	27.7	31.6	29.4	1.52	0.327	0.139	0.660
min/kg DMI	378	361	383	334	17	0.074	0.531	0.381
min/kg NDFI	1103	1124	1085	986	51	0.453	0.141	0.254
Bouts, no./day	16.7	17.8	15.9	16.0	0.98	0.520	0.191	0.586
*Total chewing*								
Time								
min/day total	707	704	771	686	25	0.100	0.376	0.124
min/kg DMI	563	525	599	493	28	0.020	0.944	0.247
min/kg NDFI	1642	1634	1698	1453	83	0.146	0.461	0.173
*Standing*								
Time								
min/day	219	238	216	241	16	0.193	1.000	0.855
min/day incl. chewing	234	264	239	281	19	0.074	0.567	0.754
min/bout	15.2	15.5	16.7	16.5	1.0	0.933	0.236	0.777
Bouts, no./day	14.4	15.3	13.1	14.7	0.6	0.045	0.119	0.552
*Lying*								
Time								
min/day	514	498	453	513	25	0.391	0.370	0.147
min/day incl. ruminating	974	957	925	939	18	0.935	0.082	0.403
min/bout	26.7	25.2	25.1	30.2	1.54	0.270	0.295	0.049
Bouts, no./day	19.2	20.2	18.4	17.3	1.1	0.929	0.104	0.378

^1^ Early maize silage, 50% of forage DM; ^2^ early maize silage, 100% of forage DM; ^3^ late maize silage, 50% of forage DM; ^4^ late maize silage, 100% of forage DM; ^5^ standard error of the mean. Values for I, M and I × M are *p*-values. n = 10 lambs in five pens per treatment; the pen was the experimental unit.

**Table 5:** Effects of dietary inclusion rate of maize silage (I) and maturity stage at harvest (M) on faecal particle-size distribution in lambs.

						Effects		
Item	E50 ^1^	E100 ^2^	L50 ^3^	L100 ^4^	SEM ^5^	I	M	I × M
*Particles retained on sieve, %*								
2.360 mm	0.03	0.32	0.07	0.07	0.08	0.096	0.235	0.100
1.000 mm	1.3	1.6	1.4	1.8	0.3	0.231	0.614	0.779
0.500 mm	12.1	13.0	12.8	15.4	1.0	0.092	0.143	0.406
0.212 mm	49.1	52.8	49.5	53.7	1.6	0.022	0.680	0.913
0.106 mm	24.3	22.1	25.1	19.8	1.5	0.026	0.632	0.316
Bottom bowl	13.3	10.2	11.2	9.3	1.1	0.032	0.177	0.572
Geometric mean, mm	0.26	0.26	0.25	0.25	0.012	0.791	0.704	0.991
Arithmetic mean, mm	0.33	0.34	0.32	0.33	0.012	0.486	0.566	0.855

^1^ Early maize silage, 50% of forage DM; ^2^ early maize silage, 100% of forage DM; ^3^ late maize silage, 50% of forage DM; ^4^ late maize silage, 100% of forage DM; ^5^ standard error of the mean. Values for I, M and I × M are *p*-values. Values for sieves are proportions (%) of particles retained on each sieve; n = 5 pens per treatment (one pooled faecal sample per pen).

## Data Availability

The data presented in this study are available upon reasonable request from the corresponding author.

## Abbreviations

The following abbreviations are used in this manuscript:

DM	Dry matter
CP	Crude protein
MP	Metabolisable protein
NDF	Neutral detergent fibre
ADF	Acid detergent fibre
ADL	Acid detergent lignin
iNDF	Indigestible NDF
IVOMD	In vitro organic matter digestibility
ME	Metabolisable energy
WSC	Water-soluble carbohydrates
DDGS	Dried distiller’s grains plus solubles
